# Effect of 12 weeks of aquatic strength training on individuals with multiple sclerosis

**DOI:** 10.1590/0004-282X-ANP-2020-0541

**Published:** 2022-02-21

**Authors:** Claudio SCORCINE, Stefanie VERÍSSIMO, Angela COUTO, Fabricio MADUREIRA, Dilmar GUEDES, Yara Dadalti FRAGOSO, Emilson COLANTONIO

**Affiliations:** 1 Universidade Federal de São Paulo, Departamento de Educação Física, Santos SP, Brazil.; 2 Universidae Metropolitana de Santos, Departamento de Educação Física e Neurociências, Santos SP, Brazil.

**Keywords:** Esclerose Múltipla, Ambiente Aquático, Treinamento de Força, Multiple Sclerosis, Aquatic Environment, Resistance Training

## Abstract

**Antecedentes:**

Programas de exercícios físicos são recomendados para pacientes com esclerose múltipla. No entanto, são limitados os estudos que envolvem o treinamento aquático de força para a melhoria das capacidades funcionais.

**Objetivo:**

Investigar o efeito de um programa de treinamento aquático de força nas capacidades funcionais e nos níveis de força e fadiga de pessoas diagnosticadas com esclerose múltipla.

**Métodos:**

Foram selecionados 29 voluntários com esclerose múltipla. Todos os participantes realizaram uma bateria de testes, incluindo os de capacidades funcionais, nível de força e níveis de fadiga em dois momentos distintos: pré-intervenção e pós-intervenção. O programa de treinamento de força foi realizado durante 12 semanas. Foram utilizados exercícios de força localizados, com controle específico de carga de trabalho, que variou entre 50 e 90% do máximo, de acordo com a semana de treinamento. Para a análise estatística, optou-se por utilizar o teste *t* de Student na comparação ente os momentos pré- e pós-intervenção.

**Resultados:**

Os resultados demonstraram melhora significativa em todas as variáveis investigadas: teste de 6 min de caminhada (p=0,00); força mão dominante (p=0,02); força mão não dominante (p=0,00); levantar (p=0,00); sentar e levantar-se (p=0,00); subir 15 degraus (p=0,00); descer 15 degraus (p=0,00); calçar meias (p=0,00); gravidade da fadiga (p=0,01); impacto da fadiga (p=0,01).

**Conclusão:**

O treinamento aquático de força foi eficiente para melhorar as capacidades funcionais relacionadas à qualidade de vida de pacientes com esclerose múltipla.

## Abstract (pt)

**Background:**

Physical exercise programs are recommended for patients with multiple sclerosis (MS). However, studies involving aquatic strength training to improve functional capacity in MS are limited.

**Objective:**

To investigate the effectiveness of an aquatic strength-training program in improving physical function, strength, and fatigue levels in individuals with (MS).

**Methods:**

Twenty-nine patients with MS were enrolled in the study. All participants underwent a battery of tests to measure physical function, strength and fatigue levels at two time points: before and after the 12-week intervention. The training program included strength exercises with clearly defined load and percentage of repetitions based on the patient’s maximal performance. Statistical analyses were carried out using the Student’s T test for comparison between pre- and post-intervention measures.

**Results:**

All test results were improved significantly after the intervention: 6-minute walk time (p=0.00), dominant hand grip strength (p=0.02), non-dominant hand grip strength (p=0.00), getting up (p=0.00), sitting and getting up (p=0.00), walking up 15 steps (p=0.00), walking down 15 steps (p=0.00), putting on socks (p=0.00), severity of fatigue (p=0.01), and impact of fatigue (p=0.01).

**Conclusion:**

The aquatic strength-training program was effective in increasing physical capacities of patients with MS.

## INTRODUCTION

Multiple sclerosis (MS) is a chronic autoimmune disease of the central nervous system, leading to inflammation, degeneration and, ultimately, persistent disability in affected patients^1^. The chronic and recurrent demyelination in MS evolves with axonal and neuronal loss and dysfunction of electrical nerve impulse transmission^2^. MS prevalence varies worldwide, with a typical latitudinal grading in which the disease is more common away from the equatorial line^3^. MS prevalence in Brazil follows this gradient pattern with 1.36 cases/100,000 inhabitants in the Northeast to 27.2/100,000 inhabitants in the South of the country^4^. The disease typically affects women between 20 and 40 years of age, and presents with a wide range of neurological symptoms. These symptoms may recover, recur, or become disabilities. Among these signs and symptoms are motor and sensory deficits, tremors, decreased coordination, visual impairment, and sphincter dysfunction^5^. Mood and sleep disorders, cognitive dysfunction, and fatigue are also significant in the lives of people with MS^6^.

Measuring disability in MS is challenging. Given the wide variety of signs, symptoms, and comorbidities that affect patients, it is virtually impossible to measure the impact of disabilities. No scale covers all possible dysfunctions and their impact, and neurologists have agreed to maintain the Expanded Disability Scale Score (EDSS)^7^ as the standard measure in studies. The EDSS is graded in half points from zero (normal) to 10 (MS-related death), it is highly dependent on motor function and gait, and increased EDSS scores can associated with higher levels of fatigue^8^.

 Physical activity programs have shown to positively influence the lives of patients with MS^9^ ^,^ ^10^, especially by reducing fatigue levels. Although a variety of programs have been proposed, strength training programs show the greatest benefit for people with MS. Patients can improve strength, reduce fatigue, decrease disease progression, and have a higher quality of life^11^ ^,^ ^12^ ^,^ ^13^. Aquatic training has been highly recommended for individuals with MS^14^ ^,^ ^15^, and aerobic aquatic training programs have shown benefits for these patients since the 1980s^16^.

Recent studies have demonstrated that aerobic aquatic training programs improves walking ability and ability to get up from a sitting position^17^. In a systematic review, a significant increase in quality of life levels was observed in MS patients after aerobic aquatic training^18^. These findings demonstrate that prescribing exercise programs is a non-pharmacological way to improve physical performance and quality of life. Despite the findings in the literature on the benefits of aquatic aerobic training, a determining variable for maintaining of functional capacities is strength, and there is currently a gap regarding the benefits of aquatic strength training and the form of individualized prescription of these model-training programs for patients in different conditions.

The aim of the present study was to investigate the effects of associating an aquatic and strength training program specially designed for individuals with MS.

## METHODS

### Subjects

The Ethics and Research Committee of the Federal University of São Paulo approved the current project. A convenience sample of 26 patients with MS diagnosed and treated in the coastal region of the state of São Paulo, Brazil, was enrolled. At the time of the project, no patient exercised regularly and a team of neurologists followed all volunteers. The patients enrolled voluntarily and the group consisted of 22 women and seven men. Participants were medically examined prior to participation. Maximum disability at the time of enrollment was defined as EDSS<6.0 (able to walk even if the aid of a cane was required). All volunteers signed a free informed consent form before entering the trial.

### Materials, participants and procedure

Body weight and height were used to calculate the body mass index (BMI). Percent body fat was calculated through a standard protocol^19^.

### Physical tests

The tests were performed in two distinct time points: before and after a 12-week intervention program of aquatic strength training. The tests consisted of:

- Aerobic performance: assessed by the six-minute walk test performed in a 30-meter hall with a demarcation every three meters and two cones at the ends^20^ ^,^ ^21^ ^,^ ^22^.
- Strength of upper limbs: assessed by the handgrip test. Subjects were evaluated seated in a chair with the spine erect, knees bent at 90 degrees and shoulders in an anatomical position. The elbow remained flexed at 90 degrees, with the forearm in a neutral grip position, with the possibility to bend it up to 30 degrees. The subject was then instructed to grasp the dynamometer as hard as possible for five seconds. The test is repeated three times with a 1-minute interval between each repetition. The highest value obtained for the dominant and non-dominant hand was recorded^23^ ^,^ ^24^.
- Activities of daily living: these were assessed by getting up from the floor; sitting and getting up from a chair and walking a short distance; walking up and down 15 steps; and putting on socks^25^.
- Fatigue: assessed by the Impact of Fatigue and Severity of Fatigue questionnaires^26^.

### Aquatic strength training program

**Figure 1.**
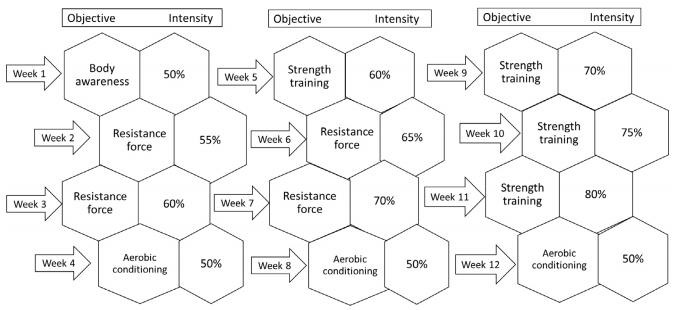
Load control model for all the exercises applied in series of 30 seconds. Repetitions were established based on the number of maximum repetitions performed in 30 seconds. The model includes a weekly goal and intensity performed by patients.

The protocol consisted of three times a week frequency for 12 weeks with 50 minutes per session. Five minutes of warm-up with aerobic and coordination exercises, forty minutes of strength training exercises. The routine was split into: Monday - exercises for the front part of the body, Wednesday - exercises for the back part of the body. In the last five minutes the subjects performed cool-down exercises. The program was performed in a 25-m swimming pool, with a water depth of 1.5 m and average temperature of 29°C, located in the Universidade Metropolitana de Santos. The load control for the strength exercises was made by the number of repetitions in a 30-second time test; the load used for the training sessions during the 12 weeks was between 50 and 80% of the maximum. The load control followed a non-linear periodization, increasing the load in three weeks and decreasing it in the fourth week (Figure 1). The interval between sets was 30 seconds, with cyclic exercises. The training protocol consisted of eight exercises, four exercises for the upper body and four exercises for the low body.

All training sessions were prescribed and accompanied by two physical education instructors.

### Statistical analysis

After confirming that the data was normally distributed with the Shapiro-Wilk test, the Student’s *t*-test was used to compare the pre- and post-intervention time points. Cohen’s test was used for assessing the effect size. The level of significance was set at p≤0.05 with a 95% confidence interval (95%CI).

## RESULTS

Anthropometric characteristics of all patients and their degree of disability are shown in Table 1. Women had higher body fat levels, as well as higher number of relapses and disability.

**Table 1.** Physical characteristics of volunteers with multiple sclerosis (mean±SD).

Characteristic	Female	Male	General
EDSS	2.2±1.3	1.8±1.5	2.1±1.3
Number of relapses	3.8±5.4	1.5±1.9	3.4±4.9
Weight (kg)	72.5±17.7	74.8±13.5	72.9±16.7
Height (cm)	1.67±0.1	1.78±0.1	1.65±0.1
BMI	27.4±6.2	23.4±3.0	26.6±5.9
% body fat	31.4±6.3	17.4±5.2	28.5±8.3
Weight body fat (kg)	23.6±10.0	13.5±5.5	21.5±10.1
Lean muscle mass (kg)	48.3±8.8	61.1±8.1	51.0±10.0

SD: standard deviation; EDSS: Expanded Disability Scale Score; BMI: body mass index.

Table 2 shows the results of physical tests before and after the training program.

**Table 2.** Results (mean±SD), absolute difference, relative difference and comparison between the pre- and post-intervention time points for all tests.

	Pre-int.	Post-int.	Abs	Rel	p-value	Cohen’s d
6-minute walk (meters)	478.3±117.3	557.4±119.3	79.1±68.7	19%	0.00	0.66
Dom. Hand Grip (lb)	67.1±24.9	70.3±22.5	3.2±6.5	8%	0.02	0.13
Non. D. Hand Grip (lb)	60.4±19.9	65.7±17.3	5.2±7.5	12%	0.00	0.28
Getting up (seconds)	6.9±4.4	4.4±2.5	2.4±2.8	-26%	0.00	0.70
Sit and get up (seconds)	52.8±19.6	37.2±15.3	15.5±9.4	-29%	0.00	0.89
Up15 steps (seconds)	10.3±4.7	8.4±4.1	1.8±2.0	-16%	0.00	0.42
Down 15 steps (seconds)	10.7±6.2	8.2±4.9	2.5±2.2	-22%	0.00	0.45
Putting socks (seconds)	13.9±9.4	10.8±8.1	3.1±4.8	-20%	0.00	0.35
Severity of fatigue (points)	39.2±18.3	32.1±16.8	7.5±13.0	-12%	0.01	0.40
Impact of fatigue (points)	47.7±19.6	38.4±21.8	9.3±16.2	-20%	0.01	0.44

SD: standard deviation; Pre-int.: pre-intervention; Post-int.: post-intervention; Abs: absolute difference; Rel: relative difference; 6-minute walk: 6-minute walk test in meters; Dom. Hand Grip: dominant hand grip test in Newton’s; Non. D. Hand Grip: non-dominant hand grip test in Newton’s; Get up: getting up from the floor; sit and get up: sitting and getting up from a chair and moving a short distance; Up15 steps: walking up 15 steps; Down 15 steps: walking down 15 steps in seconds; Putting socks: putting on socks in seconds; Impact of fatigue: impact and severity of fatigue in scores.

## DISCUSSION

In the present study, volunteers significantly improved their performance in all tests and reduced the time required to perform all functional tests (getting up from the floor, sitting and getting up from a chair and walking a short distance, walking up 15 steps, walking down 15 steps, and putting on socks). Fatigue levels decreased. There was an improvement in handgrip strength, which relates to upper limb strength and is extremely important for the patient to perform daily tasks such as carrying objects. For the physical performance tests, the improvements varied from 16 to 29%. These tests are related to the activities of daily living, such as getting up from a chair and picking up an object in another room, which are closely associated with patients’ physical independence, and therefore, with their quality of life levels.

The decrease in fatigue levels post-intervention is extremely important, since fatigue is present in most patients with MS and is one of the main disabling factors of the disease. Finally, patients increased the distance covered in the six-minute walk test. This test is commonly used to measure the aerobic conditioning and gait mobility of patients, and findings demonstrated that even though the patients performed aquatic strength training, their aerobic fitness was also improved. Taken together, the results confirm that aquatic strength training can be a non-pharmacological alternative for patients with MS.

The results of the six-minute walk test are similar to those of other studies^20^ showing the beneficial effects of an aquatic training program. The good response of strength training programs are related to the activation of upper agonist muscles and of lower antagonist muscles^27^ ^,^ ^28^. As previously shown by other authors, our results indicate that higher strength levels may ultimately be responsible for improved walking capacity and decreased fatigue levels^11^ ^,^ ^15^ ^,^ ^29^. Strength training with weights showed similar improvements to the present study. A study investigating direct and contralateral strength training in patients with multiple sclerosis for 6 weeks found 16.5% improvement in walking speed^30^, data very similar to the present study, which found a 17% improvement in walking speed. A recent study showed improvement in the ­walking speed after 24 weeks of strength training combined with cognitive exercises, corroborating the present findings in the 6-minute walk test^31^.

The high level of fatigue common in patients with MS may limit daily activities. Apart from a small effect of amantadine in some cases, no drug intervention seems to improve this situation^32^. On the other hand, aerobic training and strength training programs seem to positively affect this disabling MS symptom^33^ ^,^ ^34^ ^,^ ^35^. Two recent systematic reviews demonstrated the benefits of this type of programs in patients with MS^11^ ^,^ ^15^. The aquatic strength training is safe and effective in MS, since high intensity training can be performed with minimal risk of lesions^35^, low post-exercise pain^36^, and better control of body temperature^37^. High intensities on aquatic strength training promote an increase in fatigue tolerance and a decrease in perceived effort in daily activities^34^.

In conclusion, aquatic strength training is an effective non-pharmacological strategy to increase physical functional capacities associated to quality of life of patients with MS. The practical applications of this study is that physicians accompanying patients with multiple sclerosis can include guided aquatic strength training in their prescriptions.
